# SpatialDWLS: accurate deconvolution of spatial transcriptomic data

**DOI:** 10.1186/s13059-021-02362-7

**Published:** 2021-05-10

**Authors:** Rui Dong, Guo-Cheng Yuan

**Affiliations:** 1 Department of Pediatric Oncology, Dana-Farber Cancer Institute, Harvard Medical School, Boston, MA 02215 USA; 2 Massachusetts General Hospital Cancer Center, Harvard Medical School, Charlestown, MA 02129 USA; 3 Department of Genetics and Genomic Sciences, Charles Bronfman Institute for Personalized Medicine, Icahn School of Medicine at Mount Sinai, New York, NY 10029 USA

**Keywords:** Spatial transcriptomics, Single cell, Deconvolution

## Abstract

Recent development of spatial transcriptomic technologies has made it possible to characterize cellular heterogeneity with spatial information. However, the technology often does not have sufficient resolution to distinguish neighboring cell types. Here, we present spatialDWLS, to quantitatively estimate the cell-type composition at each spatial location. We benchmark the performance of spatialDWLS by comparing it with a number of existing deconvolution methods and find that spatialDWLS outperforms the other methods in terms of accuracy and speed. By applying spatialDWLS to a human developmental heart dataset, we observe striking spatial temporal changes of cell-type composition during development.

**Supplementary Information:**

The online version contains supplementary material available at 10.1186/s13059-021-02362-7.

## Background

Rapid development in spatial transcriptomics has enabled systematic characterization of cellular heterogeneity while preserving spatial context [1–6]. Compared to the commonly-used single-cell RNA-seq technology, the main advantage of spatial transcriptomic technologies is that they can be used to profile gene expression in a small number of or even single cells while preserving spatial information. This is crucial for mapping the structural organization of tissues and facilitates mechanistic studies of cell-environment interactions. On the other hand, identifying the spatial distributions of various cell types can be challenging, since many existing methods do not have single-cell resolution, such as Spatial Transcriptomics [4], 10X Genomics Visium, Slide-seq [2], DBiT-seq [6], and Nanostring GeoMx. This is an important barrier for data analysis and interpretation which limits the utility of these technologies. Therefore, it is desirable to develop computational methods to infer the composition of cell types at each location, a task that is often referred to as cell-type deconvolution.

A number of methods have been developed for deconvolving bulk RNAseq data [7–13]. In principle, these methods can be directly applied to spatial expression analysis as well, treating the data from each location as a bulk sample. However, there are two main limitations for this approach. First, the number of cells within each spot is typically small. For example, each spot in the 10X Genomics Visium platform has the diameter of 55 μm, corresponding to a spatial resolution of 5–10 cells. The application of a bulk RNAseq deconvolution method to such a small sample size would result in noise from unrelated cell types. Second, as spatial expression datasets usually contain thousands of spots, it would be time and memory consuming if deconvolution methods designed for bulk RNA-seq are applied on spatial expression datasets. Recently, several methods have been developed specifically for spatial transcriptomic data deconvolution [14–16]. Here, we introduce a novel method spatialDWLS for this task and benchmark with existing methods.

## Results

### Overview of spatialDWLS

**Fig. 1:**
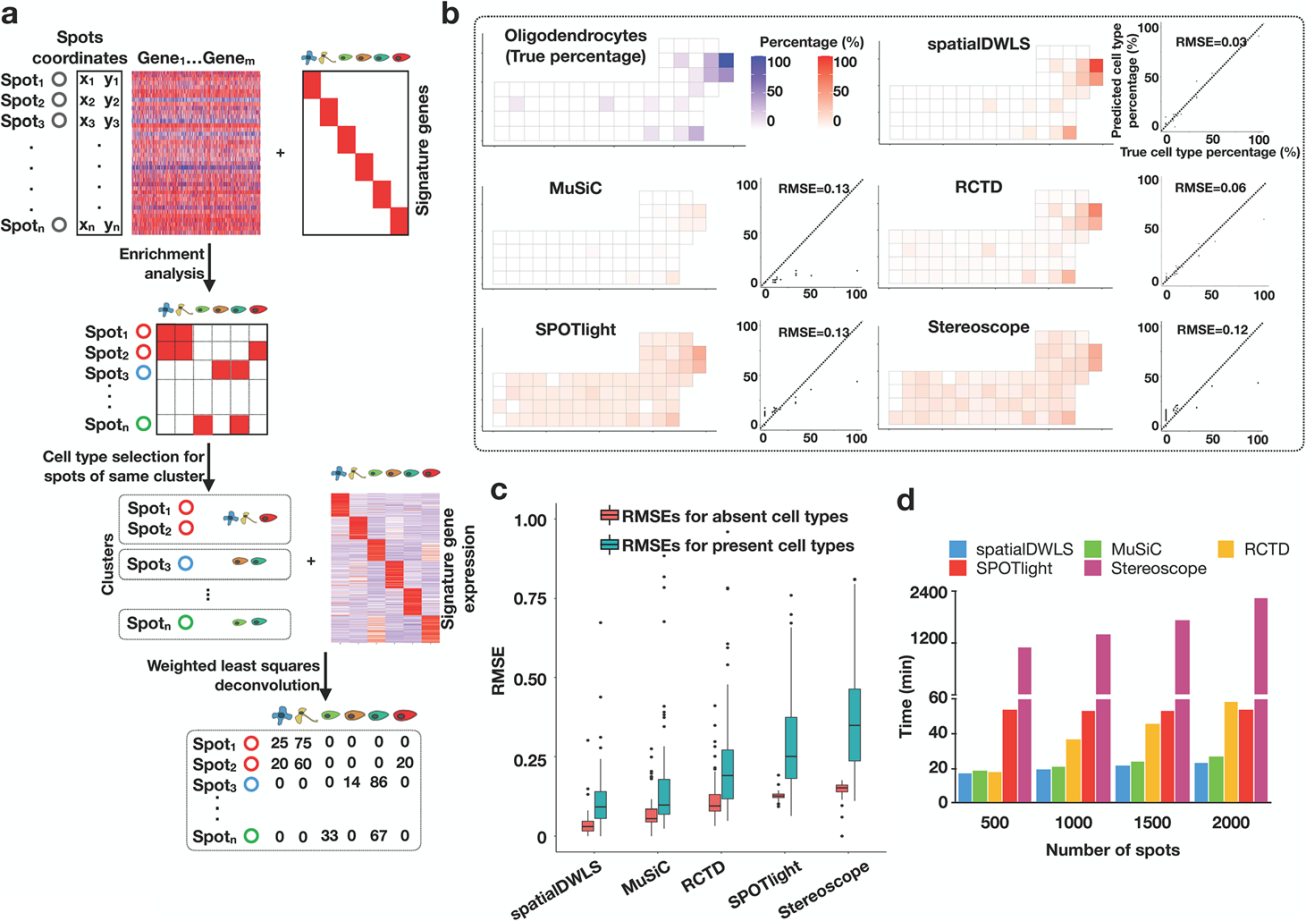
An overview of the spatialDWLS method. **a** A schematic representation of the spatialDWLS workflow. The input contains a spatial transcriptomic dataset (gene expression matrix and cell location coordinates) and a set of known cell-type specific gene signatures. For each spot, the cell types that are likely to be present are identified by using cell-type enrichment analysis. Then, a modified DWLS method is applied to infer cell type position at each spot. **b** Comparison of the accuracy of different deconvolution methods. Single-cell resolution seqFISH+ data are coarse-grain averaged to generate lower-resolution spatial transcriptomic data. The true frequency of a cell-type (indicated as blue squares in the top left panel) at each spot is compared with the inferred frequency (indicated as red squares in the five other panels) by using different methods. The relationship is also represented as a scatter plot, with *x*-axis representing the true frequency and the *y*-axis representing the inferred frequency. The overall performance is quantified as the root mean square error (RMSE). The oligodendrocyte cell-type is used here as a representative example. **c** The overall RMSE error is further decomposed into two components, corresponding to regions where the cell type is absent (red) and present (green), respectively. **d** Comparison of the computing speed of different methods. Running times for analyzing a mouse brain Visium dataset are shown

SpatialDWLS is an extension of dampened weighted least squares (DWLS) [9], which we developed previously for deconvolution of RNAseq data. In short, DWLS uses a weighted least squares approach to infer cell-type composition, where the weight is selected to minimize the overall relative error rate. Because only a small number of cell types may be associated with a specific location, we combine a recently developed cell-type enrichment analysis method [17] to enhance specificity. In a nutshell, spatialDWLS contains two steps (Fig. 1a). First, it identifies cell types that likely to be present at each location [17]. Second, the cell type composition at each location is inferred by applying DWLS to infer the fraction of each selected cell type [9]. The details are described in the “Methods” section.

### Evaluation and benchmarks of spatialDWLS

To evaluate the performance of spatialDWLS, we created a simulated spatial transcriptomic dataset based on coarse-graining average of single-cell resolution data. Specifically, we analyzed a public seqFISH+ dataset [1], which contains the expression profile of 10,000 genes in 523 cells from the mouse somatosensory cortex at the single-cell resolution. To mimic the outcome of a lower-resolution profiling strategy, we divided each field of view (FOV) into squared spots of ~ 51.5 μm on each side and aggregated the transcript counts that fell into each spot. On average, about seven cells are included in each spot. The resulting dataset has a total number of 71 spots, each covering an average of 7.3 cells. The original dataset serves as the ground-truth for benchmarking.

To apply spatialDWLS, we obtained cell-type specific gene signatures from a publicly available single-cell RNAseq (scRNAseq) dataset [18]. In total, this dataset contains 1691 cells and 6 major cell types are identified. Based on the scRNAseq derived cell-type gene signatures, we applied spatialDWLS to deconvolve the above simulated dataset. The cell-type percentage at each location varies from 5.9 to 100%.

To evaluate the performance of our spatialDWLS method, we compared the predicted and true cell type proportion and found good agreement overall (Fig. 1b–c and Additional file 1: Figure S1a-b). For example, the root mean square error (RMSE) associated with oligodendrocytes is only 0.03 with the predicted values approximately center around ground-truth (Fig. 1b). In order to separately evaluate the sensitivity and specificity, we divided the simulated spots into subsets where the cell type was present or absent and evaluated the RMSE errors for each subset. The fact that both errors have small magnitude indicates spatialDWLS has both high degrees of sensitivity and specificity (Fig. 1c). As a benchmark, we applied four published deconvolution methods, including MuSiC [8], RCTD [14], SPOTlight [15], and stereoscope [16] to analyze the same dataset. All the other methods led to higher error (Fig. 1b, Additional file 1: Figure S1a, b), although the differences with MuSiC and RCTD appear modest.

Next, we applied spatialDWLS to analyze a 10X Genomics Visium dataset mapping the spatial transcriptomic profile in mouse brain. This dataset contains 2698 spatially barcoded circular spots each 55 μm in diameter. To comprehensively deconvolve cell type composition, we used the mouse nervous system atlas scRNAseq data as a reference [19], which contains gene expression signature of 21 major cell types. While it is impossible to quantify the prediction accuracy because the ground-truth is unknown, the resulting spatial distributions are highly consistent with the mouse Allen Brain Atlas (Additional file 1: Figure S2a, b). For example, the peptidergic cells were correctly mapped to the hypothalamus region; the granule neurons were correctly mapped to the dentate gyrus region, and the medium spiny neurons were correctly mapped to the basal ganglia (Additional file 1: Figure S2a, b).

The spatialDWLS analysis took 23 min CPU time on a small computer cluster (Intel Xeon CPU E5-2650 32 processors 2.00GHz and 380Gb memory). To compare the computational efficiency of different methods, we applied each of the other methods to analyze the same dataset using the same computer. Furthermore, to assess scalability we subsampled the mouse brain dataset varying from 500 to 2000 spots and examined the relationship between CPU time and sample size. We found that spatialDWLS and MuSiC were more computationally efficient, each taking about 23 min CPU time to analyze the 2000-spot dataset. In comparison, both RCTD and SPOTlight were about 2 times slower for the larger sample size, whereas stereoscope was at least 10 times slower (Fig. 1d). Taken together, these analyses suggest spatialDWLS is more accurate and computationally efficient than these other methods.

### Apply spatialDWLS on human heart spatial transcriptomic dataset

**Fig. 2:**
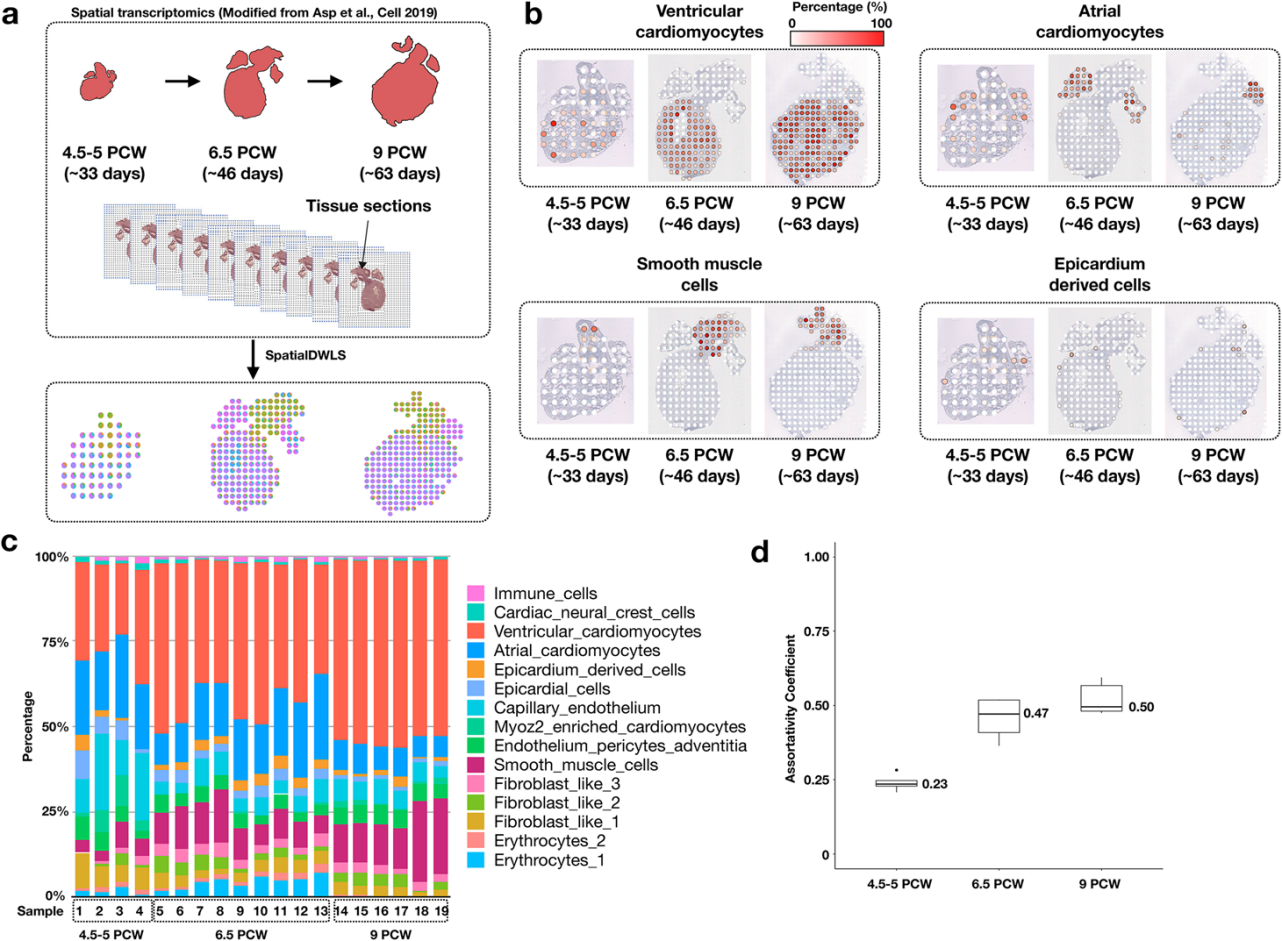
Deconvolution analysis identifies spatial-temporal change of cell-type composition during human heart development. **a** A schematic overview of the analysis. Spatial Transcriptomic data for developing heart were collected at three developmental stages by Asp et al. 2019. In parallel, single-cell RNAseq analysis was carried out to identify cell-type specific gene signatures. The spatialDWLS method was applied to infer the distribution of different cell-types across developmental stages. **b** The resulting estimates of the spatial distribution of different cell types. One representative sample was selected from each developmental stage. **c** A summary of the cell-type composition for all samples grouped by the corresponding developmental stages. **d** The assortativity analysis indicates an increased level of spatial clustering among similar cell types during heart development

During embryonic development, the spatial-temporal distribution of cell types changes dramatically. Therefore, it is of interest to test whether spatialDWLS could aid the discovery of such dynamic changes. Recently, Asp and colleagues studied the development of human heart in early embryos (4.5–5, 6.5, and 9 post-conception weeks) by using the Spatial Transcriptomics (ST) technology [20] (Fig. 2a). Since the data does not have single-cell resolution, they were not able to identify cell-type distribution directly from the ST data. In order to apply spatialDWLS, we utilized the scRNAseq derived gene signatures from this study as reference. All the cell types were mapped to expected locations (Fig. 2b and Additional file 1: Figure S3a-c).

In order to quantitatively compare the change of spatial-temporal organization of cell type composition during embryonic heart development, we first examined the overall abundance of different cell types (Fig. 2c). We found that the abundance of ventricular cardiomyocytes increases dramatically during development (from an average of 25% in weeks 4.5–5 to 53% in week 9) (Fig. 2c). Notably, the abundance of atrial cardiomyocytes does not show this trend, which probably reflects the atrium compartments expand less dramatically compared to the ventricle compartments. Next, we compared the spatial organization patterns across developmental stages. Normal heart function relies on the coordinated activity of billions of cardiac cells; therefore, we were interested to test whether spatially neighboring cells tend to belong to the same cell type. This is quantified by using a metric called the assortativity coefficient [21], which is commonly used in social network analysis to characterize the tendency of friendship formed by similar individuals. In the current context, we considered the spatial network connecting neighboring cells. We further modified the definition of assortativity coefficient in order to account for the cellular heterogeneity within each spot location (see the “Methods” section for details). We found that the assortativity coefficient increased from 0.23 at weeks 4.5–5 to 0.50 at week 9 (Fig. 2d), suggesting the spatial organization becomes increasingly spatially coherent during heart development.

## Conclusions

SpatialDWLS is an accurate and computationally efficient method for estimating the spatial distribution of cell types from spatial transcriptomic data. Thus, it provides a valuable enabling toolkit for investigating cell-cell interactions from various spatial transcriptomic technology platforms that do not have single-cell resolution. Compared to existing deconvolution methods [7–16], the key difference is spatialDWLS contains an additional filtering step to remove irrelevant cell-types thereby enhancing specificity. The spatialDWLS method can be easily accessed in Giotto [17], which is a user-friendly software package containing a large number of computational tools for spatial transcriptomic data analysis and visualization.

## Methods

### Cell type selection of spatial expression data by enrichment analysis

We use an enrichment based weighted least squares approach for deconvolution of spatial expression datasets. First, enrichment analysis using Parametric Analysis of Gene Set Enrichment (PAGE) method [22] is applied on spatial expression dataset as previously reported [17]. The marker genes can be identified via differential expression gene analysis of Giotto based on the scRNAseq data provided by users. Alternatively, users can also provide marker gene expression for each cell type for deconvolution. The number of cell-type specific marker genes is denoted by *m*. For each gene, we calculate the fold change as the ratio between its expression value at each spot and the mean expression of all spots. The mean fold change of the *m* marker genes is calculated and denoted as *S*_*m*_. As background control, the mean and standard deviation of the fold change values across all genes are denoted as μ and δ, respectively. The enrichment score (ES) is defined as follows:

$$ \mathrm{ES}=\frac{\left({S}_m-\mu \right)\ast \sqrt{m}}{\delta } $$

Then, we binarize the enrichment matrix with the cutoff value of ES = 2 to select cell types that are likely to be present at each point.

### Estimating cell type composition by using a weighted least squares approach

In previous work, we developed DWLS [9] for deconvolution of scRNAseq data. This method is extended here to deconvolve spatial transcriptomic data using the signature gene identification step described above. In short, DWLS uses a weighted least squares approach to infer cell-type composition, where the weight is selected to minimize the overall relative error rate. In addition, a damping constant *d* is used to enhance numerical stability, whose value is determined by using a cross-validation procedure. Here, we use the same sets of weights and damping constant across spots within same clusters to reduce technical variation. Finally, since the number of cells present at each spot is generally small, we perform another round deconvolution after removing those cell types that are predicted to present at a low frequency by imposing an additional thresholding (min frequency = 0.02 by default).

### Coarse-grained spatial transcriptomic data for model performance evaluation

The somatosensory cortex seqFISH+ data were abstained from https://github.com/CaiGroup/seqFISH-PLUS. To simulate spot-like data, we defined the square with 500 pixels time 500 pixels (~ 51.5 μm) as one spot-like region. Then, average expression level was calculated for each spot-like region. Due to the small sample size, we only considered the 6 major clusters: excitatory neurons (eNeuron), inhibitory neurons (iNeuron), astrocytes, oligodendrocytes (Olig), microglia cells, and endothelial-mural cells (endo_mural).

### Benchmark comparison among different methods

Coarse-grained seqFISH+ dataset was used for benchmarking the accuracy of different deconvolution methods, including spatialDWLS, MuSiC, RCTD, SPOTlight, and stereoscope. For each published method, the default parameter setting was used for comparison. If the users are required to set parameters manually, we used the values suggested in the vignettes of the corresponding software. Cell-type annotations for the original, single-cell resolution data were used as the ground-truth. All five methods used the same scRNA-seq dataset as a reference in deconvolution.

For spatialDWLS, we clustered the spot-like regions by using Leiden clustering as implemented in Giotto (Version 1.0.3) by using the following commands *createNearestNetwork(dimensions_to_use = 1:10, k = 4)* and *doLeidenCluster(resolution = 0.4, n_iterations = 1000)*.

Then, marker genes of major clusters were identified by using the *findMarkers_one_vs_all* function with parameter setting: *method = ‘gini’, expression_values = ‘normalized’*. Top 100 ranked genes for each cell type were selected as marker genes. Average marker gene expression was calculated based on the cell type annotation of scRNA-seq. Then, deconvolution was applied by using the *runDWLSDeconv* function.

MuSiC [8] (version 0.1.1) was used for deconvolution by using whole single cell RNA-seq matrix. *ExpressionSet* classes were generated for both single cell RNA-seq (*SC.eset*) and spatial expression datasets (*ST.eset*). Then, cell type proportion was estimated by using *music_prop(bulk.eset = ST.eset, sc.eset = SC.eset).*

Then, to perform deconvolution by using SPOTlight [15] (version 0.1.0), signature genes were identified based on the major cell type annotation by using *Seurat::FindAllMarkers(logfc.threshold = 1, min.pct = 0.9).*

Deconvolution was performed by using *spotlight_deconvolution(se_sc = SC, counts_spatial = ST, cluster_markers = cluster_markers_all, clust_vr = “label”).*

Next, we used stereoscope [16] (version 0.2.0) for the deconvolution of simulated dataset. Deconvolution was performed with the parameter: *stereoscope run -scc SC.tsv -scl cell_labels.tsv -stc ST.tsv -sce 5000.*

Finally, we used RCTD [14] (version 1.1.0) to evaluate the cell type composition for simulated seqFISH+ dataset. Signature genes were identified by using “*dgeToSeurat*,” and then “*create.RCTD*” and “run.RCTD” were used to decompose the cell type composition. Finally, cell type percentage for each spot was calculated using the “*sweep*” function.

The computational efficiency of different methods was benchmarked by using the Visium brain dataset. All analyses were done on the same computer, which had Intel Xeon CPU E5-2650 2.00GHz and 380Gb memory. Of note, the Visium data cannot be used to evaluate accuracy because the ground-truth is not known.

### Root mean square error (RMSE) calculation

Based on the cell type annotation of seqFISH+ dataset, we calculated the true cell type percentage for simulated spatial expression datasets. For a specific cell type, we divided spot-like regions into two groups based on the presence or absence of this cell type. RMSEs were calculated separately for these two groups.

### Analysis of a spatial transcriptomic dataset from the mouse brain

The Visium dataset was obtained from the 10X Genomics website (https://support.10xgenomics.com/spatial-gene-expression/datasets/1.1.0/V1_Adult_Mouse_Brain), which corresponds to a coronal section of the mouse brain. Then, Giotto was used for data analysis as (http://www.spatialgiotto.com/giotto.visium.brain.html). Only spots within tissue were kept for further analysis. Then, we filtered out low quality spots and genes by using filterGiotto with the parameter: *expression_threshold = 1, gene_det_in_min_cells = 50, min_det_genes_per_cell = 1000*.

After normalization and highly variable gene calculation, we performed neighborhood analysis with parameter: *createNearestNetwork (dimensions_to_use = 1:10, k = 15)* and clustered spots with the parameter: *doLeidenCluster(resolution = 0.4, n_iterations = 1000)*. Finally, we used marker genes and scRNA-seq reported in Zeisel et al. [18] to deconvolute the Visium dataset.

### Analysis of a spatial transcriptomic dataset from developing human heart

The human heart spatial transcriptomics datasets were obtained from [20]. Then, we filtered out low quality spots and genes by using filterGiotto with the parameter: *expression_threshold = 1, gene_det_in_min_cells = 10, min_det_genes_per_cell = 200*.

After normalization and highly variable gene detection, we performed neighborhood analysis with theparameter: *createNearestNetwork(dimensions_to_use = 1:10, k = 10)* and clustered spots with the parameter: *doLeidenCluster(resolution = 0.4, n_iterations = 1000)*.

In addition, we use the scRNA-seq data from the same website with spatial transcriptomics datasets. Based on the clusters reported, we re-analyzed signature genes by using Giotto with the parameter: *findMarkers_one_vs_all(method = 'scran')*.

The average expression of marker genes was used for the deconvolution of heart ST datasets.

### Assortativity analysis

To evaluate the degree of spatial coherence, we extended the assortativity analysis [21], a method commonly used in the network analysis to evaluate the tendency of similar networks nodes are connected to each other. Here, we generated a spatial network by connecting spots that are immediately next to each other. The assortativity coefficient represents the normalized deviation of edges connecting the same cell type than expected by chance. More precisely, it is defined by the following formula:

$$ Q=\frac{\sum_k{q}_{kk}-{\sum}_k{a}_k^2}{1-{\sum}_k{a}_k^2} $$

where

$$ {q}_{kk}=\frac{\sum_i{\sum}_j{w}_k^i{w}_k^j{e}_{ij}}{\sum_i{\sum}_j{e}_{ij}} $$

and

$$ {a}_k=\frac{1}{N}{\sum}_i{w}_k^i $$

In the above, ${w}_k^i$ represents the fraction of cell-type *k* at the *i*th spot, *N* represents the total number of spots, and *e*_*ij*_ is defined as

$$ {e}_{ij}=\left\{\begin{array}{c}1,\mathrm{if}\ i\ \mathrm{and}\ j\ \mathrm{are}\ \mathrm{neighboring}\ \mathrm{spots}\\ {}0,\mathrm{otherwise}\end{array}\right. $$

If the values of ${w}_k^i$ are binary, then the above definition reduces to the original formula in [21].

## Supplementary Information

**Additional file 1: Figure S1-S3.**

**Additional file 2.** Review history.

## Data Availability

All codes, data, and analysis results in this paper are publicly available at GitHub [23] and at Zenodo [24]. The source code is released under the MIT license. Furthermore, the spatialDWLS method is implemented as the *runDWLSDeconv* function in Giotto (https://github.com/RubD/Giotto), and detailed tutorial and vignette are available at Giotto websites (http://www.spatialgiotto.com and https://rubd.github.io/Giotto_site/).
