# Supplementary material for: SpatialDWLS: accurate deconvolution of spatial transcriptomic data
Source: Genome Biol. 2021 May 10;22:145. doi: 10.1186/s13059-021-02362-7 (PMC8108367; doi:10.1186/s13059-021-02362-7)
Supplement: Supplementary file 1 — Additional file 1: Figure S1-S3. [file 13059_2021_2362_MOESM1_ESM.pdf]

Supplementary Figure Legends

Fig S1. Extended comparison of the accuracy of different deconvolution methods (see Fig 1b for description).

a Heatmaps indicate the spatial distribution of different cell types. The true frequency (blue) are compared with estimates from different deconvolution methods (red). The RMSE corresponding to each cell type is indicated.

b Scatter plot indicate the relationship between true (y-axis) and inferred (x-axis) cell type frequencies. Results from all the cell types are overlaid. The overall level of RMSE is indicated for each method.

Fig S2. Deconvolution analysis of the mouse brain Visium dataset.

a The spatialDWLS estimated spatial distribution of different cell types (indicated as red dots).

b. The anatomic structure of the brain, obtained from the Allen Brain Atlas, is shown here for comparison with the deconvolution analysis results.

Fig S3. Deconvolution analysis of the human developing heart Spatial Transcriptomic dataset.

SpatialDWLS estimated spatial distribution of atrial cardiomyocytes, ventricular cardiomyocytes, smooth muscle cells and epicardium derived cells in week 4.5-5 (**a**), week 6.5 (**b**) and week 9 (**c**). The magnitude of cell-type frequency at each spot is indicated by red circles.

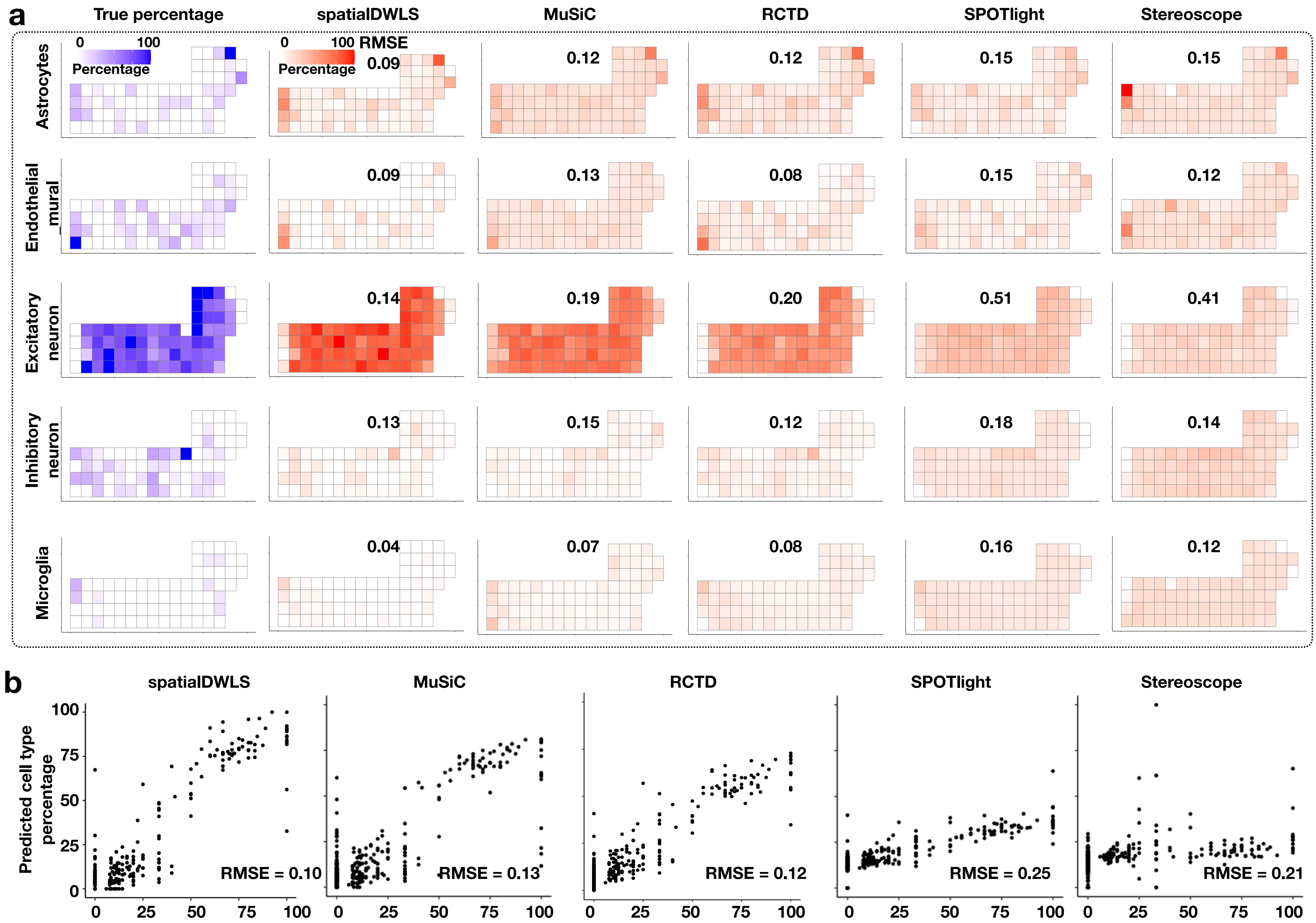

a
True percentage
spatialDWLS
MuSiC
RCTD
SPOTlight
Stereoscope
0
100
Percentage
RMSE
Astrocytes
Endothelial_mural
Excitatory neuron
Inhibitory neuron
Microglia
0.09
0.12
0.12
0.15
0.15
0.09
0.13
0.08
0.15
0.12
0.14
0.19
0.20
0.51
0.41
0.13
0.15
0.12
0.18
0.14
0.04
0.07
0.08
0.16
0.12
b
Predicted cell type percentage
True cell type percentage
RMSE = 0.10
RMSE = 0.13
RMSE = 0.12
RMSE = 0.25
RMSE = 0.21
0
25
50
75
100

Figure S1

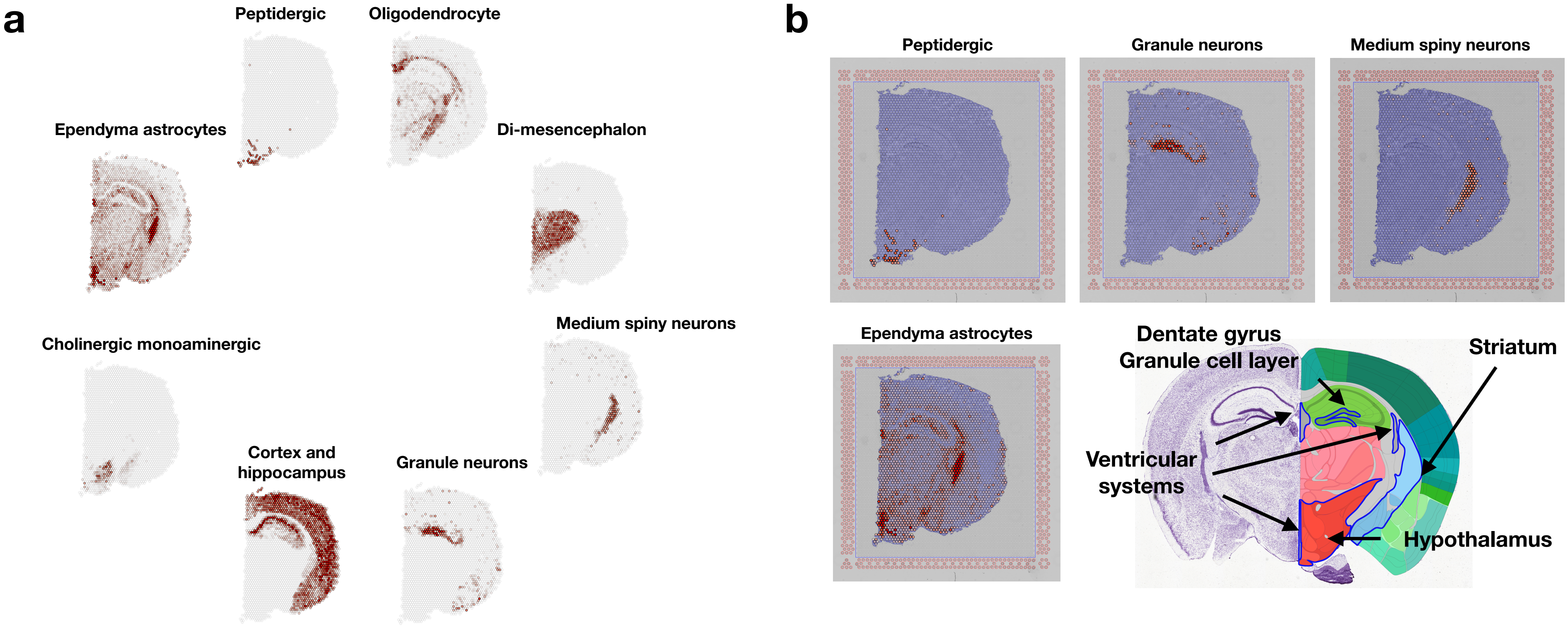
a
Peptidergic
Oligodendrocyte
Ependyma astrocytes
Di-mesencephalon
Cholinergic monoaminergic
Medium spiny neurons
Cortex and hippocampus
Granule neurons
b
Peptidergic
Granule neurons
Medium spiny neurons
Ependyma astrocytes
Dentate gyrus
Granule cell layer
Striatum
Ventricular systems
Hypothalamus

Figure S2

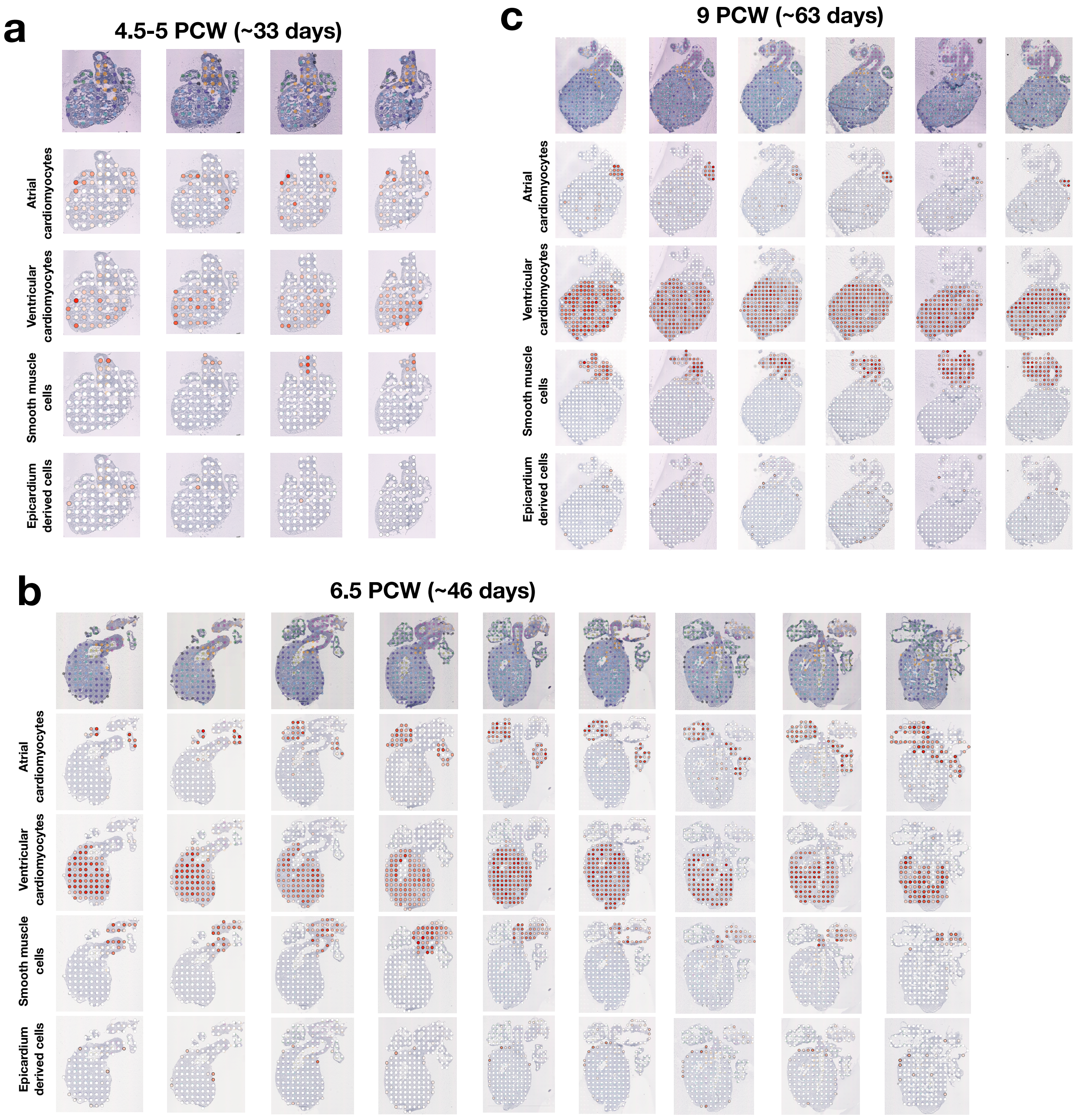
a
4.5-5 PCW (~33 days)
c
9 PCW (~63 days)
b
6.5 PCW (~46 days)
Atrial cardiomyocytes
Ventricular cardiomyocytes
Smooth muscle cells
Epicardium derived cells

Figure S3
