# Supplementary material for: SpatialDWLS: accurate deconvolution of spatial transcriptomic data
Source: Genome Biol. 2021 May 10;22:145. doi: 10.1186/s13059-021-02362-7 (PMC8108367; doi:10.1186/s13059-021-02362-7)
Supplement: Supplementary file 2 — Additional file 2. Review history. [file 13059_2021_2362_MOESM2_ESM.docx]

Review History

**First round of review**

**Reviewer 1**

**Were you able to assess all statistics in the manuscript, including the appropriateness of statistical tests used?**

Yes: Statistical methods are appropriate

**Were you able to directly test the methods?**

No

**Comments to author:**

This paper describes a new tool for spatial deconvolution of spatial genomics data when combined with single cell (or cell type signature) data to infer spatial distributions of cell types. The approach is timely as spatial transcriptomics methods are currently exploding. The use of high resolution spatial data (seqFISH) that is spatially blurred to simulated lower resolution spatial data is compelling. While there are a few other methods for this type of spatial deconvolution, the authors show that, at least on their simulated test data, their method is more computationally efficient and tends to perform better. The paper is clearly written and figures are easy to follow and appropriate for Genome Biology. I only have a few very minor suggestions.

1)	The Results is short and while very easy to read could use a bit more intuitive explanation of the steps in the method (with the details remaining in the methods section)

2)	The discussion should do more to contrast how the methods applied here differ technically from those in other previously published methods (MuSiC, SPOTlight etc) and how this might relate to the observed improvements in accuracy and computational efficiency

3)	Similarly, for a non-expert, the choice of parameters for the competing approaches could seem arbitrary. Some description of whether and how the authors attempted to optimize these parameters (and feature selection if different) is warranted - as the authors are well aware the choice of parameters can heavily influence the accuracy of these types of methods.

4)	This is optional but it seems to me that this general problem is actually closely related to cell doublet/multiplet detection in standard single cell experiments; if the authors agree it could be worth discussing potential for identification and maybe deconvolution of such multiplets.

5)	Given that there are only 2 "main" figures the Supplemental Figures could easily be converted to main text figures.

**Reviewer 2**

**Were you able to assess all statistics in the manuscript, including the appropriateness of statistical tests used?**

Yes: no additional tests required

**Were you able to directly test the methods?**

No

**Comments to author:**

Dong and Yuan present a novel method for the deconvolution of spatial transcriptomics data. Specifically, they adapted the DWLS algorithm for deconvolving bulk RNAseq data with designs to handle the issue of small cell numbers per spot in spatial transcriptomics. The manuscript is clearly written and enjoyable to read. The method is not only accurate but also computationally efficient, which should be of interest to the genomics community. Specific comments follow.

1. In terms of writing, could the authors more explicitly explain how spatialDWLS gets around the general constraint of small cell numbers?

2. In terms of the observation of increasing spatial coherence during heart development, it is interesting but somewhat counterintuitive to a developmental biologist. Could there be technical issues underlying the observation? For example, at the earlier stage the cells types are less differentiated, and less prominent differences in DEGs increase the uncertainty of deconvolution?

**Authors Response**

**Point-by-point responses to the reviewers’ comments:**

Response to Reviewer #1’s comments

This paper describes a new tool for spatial deconvolution of spatial genomics data when combined with single cell (or cell type signature) data to infer spatial distributions of cell types. The approach is timely as spatial transcriptomics methods are currently exploding. The use of high resolution spatial data (seqFISH) that is spatially blurred to simulated lower resolution spatial data is compelling. While there are a few other methods for this type of spatial deconvolution, the authors show that, at least on their simulated test data, their method is more computationally efficient and tends to perform better. The paper is clearly written and figures are easy to follow and appropriate for Genome Biology. I only have a few very minor suggestions.

*Response: We thank the reviewer for his/her positive evaluation of our manuscript and helpful suggestions. We have revised our manuscript as suggested by the reviewer as detailed below.*

1) The Results is short and while very easy to read could use a bit more intuitive explanation of the steps in the method (with the details remaining in the methods section).

*Response: We thank the reviewer for this suggestion and have added more intuitive explanation in the Results section.*

*The revised text reads:” SpatialDWLS is an extension of DWLS [9], which we developed previously for deconvolution of single-cell RNAseq data. In short, DWLS uses a weighted least squares approach to infer cell-type composition, where the weight is selected to minimize the overall relative error rate. Because only a small number of cell types may be associated with a specific location, we combine a recently developed cell-type enrichment analysis method [17] to enhance specificity. In a nutshell, spatialDWLS contains two steps (Fig 1a). First, it identifies cell types that likely to be present at each location [17]. Second, the cell type composition at each location is inferred by applying DWLS to infer the fraction of each selected cell type [9]. The details are described in the Methods section. “*

2) The discussion should do more to contrast how the methods applied here differ technically from those in other previously published methods (MuSiC, SPOTlight etc) and how this might relate to the observed improvements in accuracy and computational efficiency.

*Response: We thank the reviewer for this suggestion and added the following sentence to highlight the difference between spatialDWLS and existing methods. “Compared to existing deconvolution methods [7-16], the key difference is spatialDWLS contains an additional filtering step to filter out irrelevant cell-types thereby enhancing specificity. “*

3) Similarly, for a non-expert, the choice of parameters for the competing approaches could seem arbitrary. Some description of whether and how the authors attempted to optimize these parameters (and feature selection if different) is warranted - as the authors are well aware the choice of parameters can heavily influence the accuracy of these types of methods.

*Response: We agree the performance of a computational model may be significantly affected by its parameter setting. We did not attempt to optimize any parameters, but used the default parameter setting or parameters suggested in the vignettes of corresponding software. To make this clear, we added the following sentence in the Benchmark comparison among different methods section. “For each published method, the default parameter setting was used for comparison. If the users are required to set parameters manually, we used the values suggested in the vignettes of the corresponding software.”*

4) This is optional but it seems to me that this general problem is actually closely related to cell doublet/multiplet detection in standard single cell experiments; if the authors agree it could be worth discussing potential for identification and maybe deconvolution of such multiplets.

*Response: We thank the reviewer for this very interesting suggestion. Since similar idea has already be explored in the literature (such as DoubletDecon), we feel it is redundant to make a similar recommendation here again.*

5) Given that there are only 2 "main" figures the Supplemental Figures could easily be converted to main text figures.

*Response: The supplementary figures are used to expand the ideas and conclusions in the main figures rather than exploring new points. For this purpose, we feel the paper is more readable if we keep the supplementary figures as they are.*

Response to Reviewer #2’s comments

Dong and Yuan present a novel method for the deconvolution of spatial transcriptomics data. Specifically, they adapted the DWLS algorithm for deconvolving bulk RNAseq data with designs to handle the issue of small cell numbers per spot in spatial transcriptomics. The manuscript is clearly written and enjoyable to read. The method is not only accurate but also computationally efficient, which should be of interest to the genomics community. Specific comments follow.

*Response: We thank the reviewer for his/her positive evaluation of our manuscript and helpful suggestions. We have revised our manuscript as suggested by the reviewer as detailed below.*

1. In terms of writing, could the authors more explicitly explain how spatialDWLS gets around the general constraint of small cell numbers?

*Response: The main constraint of small cell numbers is reflected by the limited number of cell types associated with each spot. SpatialDWLS gets around this constraint by filtering out those cell types that are unlikely to be present based on cell-type enrichment analysis. This step significantly reduces the number of cell-types to be considered. To make it clearer, we expanded the Overview of spatialDWLS section by providing more intuitive explanation.*

*The revised text now reads:” SpatialDWLS is an extension of DWLS [9], which we developed previously for deconvolution of single-cell RNAseq data. In short, DWLS uses a weighted least squares approach to infer cell-type composition, where the weight is selected to minimize the overall relative error rate. Because only a small number of cell types may be associated with a specific location, we combine a recently developed cell-type enrichment analysis method [17] to enhance specificity. In a nutshell, spatialDWLS contains two steps (Fig 1a). First, it identifies cell types that likely to be present at each location [17]. Second, the cell type composition at each location is inferred by applying DWLS to infer the fraction of each selected cell type [9]. The details are described in the Methods section.”*

2. In terms of the observation of increasing spatial coherence during heart development, it is interesting but somewhat counterintuitive to a developmental biologist. Could there be technical issues underlying the observation? For example, at the earlier stage the cell types are less differentiated, and less prominent differences in DEGs increase the uncertainty of deconvolution?

*Response: We thank the reviewer for this insightful comment. We note that the single-cell RNAseq data in Asp et al. 2019 were collected from a single developmental stage (6.5-7 PCW), therefore excluding the possibility of comparing developmental-stage associated gene signature variations. The question raised by the reviewer can only be addressed in the future when more data become available.*
